# Assessment of the Mechanical Properties of Soft Tissue Phantoms Using Impact Analysis

**DOI:** 10.3390/s25051344

**Published:** 2025-02-22

**Authors:** Arthur Bouffandeau, Anne-Sophie Poudrel, Chloé Brossier, Giuseppe Rosi, Vu-Hieu Nguyen, Charles-Henri Flouzat-Lachaniette, Jean-Paul Meningaud, Guillaume Haïat

**Affiliations:** 1 Laboratoire Modélisation et Simulation Multi Echelle, Centre National de la Recherche Scientifique, MSME UMR 8208 CNRS, 61 Avenue du Général de Gaulle, 94010 Créteil, France; arthur.bouffandeau@u-pec.fr (A.B.);; 2 Laboratoire Modélisation et Simulation Multi Echelle, Université Paris Est Créteil, MSME UMR 8208 CNRS, 61 Avenue du Général de Gaulle, 94010 Créteil, France; 3 Service de Chirurgie Orthopédique et Traumatologique, Hôpital Henri Mondor AP-HP, Laboratoire IMRB INSERM U955—Université Paris—Est Créteil, 8 rue du Général Sarrail, 94010 Créteil, France; charles-henri.flouzat-lachaniette@aphp.fr; 4 Service de Chirurgie Plastique, Reconstructrice, Esthétique et Maxillo-Faciale, Hôpital Henri Mondor AP-HP, Laboratoire IMRB INSERM U955—Université Paris—Est Créteil, 8 rue du Général Sarrail, 94010 Créteil, France

**Keywords:** soft tissues, impact analysis, elastography, mechanical properties, instrumentation

## Abstract

Skin physiopathological conditions have a strong influence on its biomechanical properties. However, it remains difficult to accurately assess the surface stiffness of soft tissues. The aim of this study was to evaluate the performances of an impact-based analysis method (IBAM) and to compare them with those of an existing digital palpation device, MyotonPro^®^. The IBAM is based on the impact of an instrumented hammer equipped with a force sensor on a cylindrical punch in contact with agar-based phantoms mimicking soft tissues. The indicator Δt is estimated by analyzing the force signal obtained from the instrumented hammer. Various phantom geometries, stiffnesses and structures (homogeneous and bilayer) were used to estimate the performances of both methods. Measurements show that the IBAM is sensitive to a volume of interest equivalent to a sphere approximately twice the punch diameter. The sensitivity of the IBAM to changes in Young’s modulus is similar to that of dynamic mechanical analysis (DMA) and significantly better compared to MyotonPro. The axial (respectively, lateral) resolution is two (respectively, five) times lower with the IBAM than with MyotonPro. The present study paves the way for the development of a simple, quantitative and non-invasive method to measure skin biomechanical properties.

## 1. Introduction

Despite its empirical nature, palpation has long been used to inspect tissues’ biomechanical properties and identify potential pathologies, such as for cancer or fibrosis [1,2], which induce modifications of the mechanical properties of soft tissues [3]. In dermatology and plastic surgery, the skin biomechanical property is a marker of interest in scleroderma, keloid scars, various cancerous tumors or Ehlers–Danlos syndromes [4,5]. Additionally, the cosmetics industry is interested in the characterization of the skin firmness to assess the effectiveness of their products [6,7] and to propose a product in a customer-specific manner. A simple, reliable, objective and non-invasive method of soft tissue biomechanical characterization is likely to be used as a decision support system in various fields of medicine and cosmetics, which could motivate the development of a personalized medicine approach allowing improved diagnosis, monitoring and evaluation of therapeutic response [7,8].

Elastography emerged in the 1990s and is based on the estimation of the elastic moduli of soft tissues [9]. Transient elastography has become a reference tool in clinical practice to retrieve the soft tissue elastic modulus, like, for example, in the diagnosis of hepatic fibrosis [10,11]. However, due to its complexity and cost, and to the fact that its performances are limited when it comes to analyzing the surface of soft tissues, the use of elastography for the evaluation of skin properties remains limited. For this reason, new, relatively low-cost, easy-to-use devices have emerged. For example, the Cutometer^®^ exploits the principle of suction to exert a deformation on the skin and thus to characterize the skin superficial layers [6,7,12]. However, the method presents certain limitations in terms of reproducibility and interpretation in relation to the viscoelastic parameters of the skin [6,7]. In the same context, MyotonPro^®^ is a handheld digital palpation device for research use only, which records biomechanical parameters of soft tissues based on their response to a mechanical impulse [13,14]. This device has been used in the context of physiotherapy, neurology, dermatology or sports medicine.

Interestingly, a method based on impact analysis has recently been developed and tested with agar-based soft tissue phantoms [15]. This method derives from a technique initially developed in the framework of orthopedic surgery to assess the stability of cementless hip arthroplasty, acetabular cup [16,17,18] and femoral stem [19,20,21], based on impact analysis. A hammer instrumented with a force sensor was used to record the variation in the force as a function of time during an impact realized on the bone–implant system. The force signals were analyzed, and a time indicator was shown to be related to the implant stability. The technique was then applied to osteotomy procedures [22,23] and, more specifically, to rhinoplasty [24,25]. The impact-based analysis method (IBAM) tested with agar-based soft tissue phantoms [15] showed that a time indicator obtained from the impact signals was sensitive to the agar mass concentration. However, it was not possible to determine the mechanical properties of the samples, and the set-up suffered from reproducibility issues. Here, the experimental set-up was improved through a more reproducible phantom positioning, punch guidance and impact properties. Moreover, the sample manufacturing process has also been improved thanks to a reduction in the number of human interventions and to improved storage conditions. Moreover, the IBAM could not be compared quantitatively with other existing techniques in its ability to predict the sample Young’s modulus.

The aim of this article is to evaluate the performances (in terms of lateral and axial resolution as well as of sensitivity) of the IBAM to retrieve the soft tissue biomechanical properties and to compare them with those of the MyotonPro device. To do so, agar-based soft tissue-mimicking phantoms have been considered [26,27,28,29] with various agar mass concentrations [30,31]. The elastic moduli of the samples have been measured using a custom vibration-based set-up. The performances of both devices in terms of (i) axial and lateral resolutions and (ii) sensitivity of changes in phantoms’ Young’s modulus were compared by considering homogeneous and bilayer phantoms.

## 2. Materials and Methods

### 2.1. Sample Preparation

In order to mimic the mechanical properties of soft tissue, several phantoms made of an agar hydrogel were fabricated following Manickam et al. [26] with different stiffness values depending on the agar mass concentration comprised between 1% and 5%. To prepare the hydrogel for a mass concentration of agar $t_{ag}$, a solution containing a volume $V_{w}$ of distilled water and a mass $m_{ag}$ of agar powder was mixed in a beaker with

(1) $$m_{ag}=\frac{t_{ag}\times V_{w}\times \rho_{w}}{1-t_{ag}},$$

where $\rho_{w}$ is the density of the distilled water. The mixture was then heated using a heating magnetic stirrer. At the boiling point, heating was stopped, and the mixture was cooled while maintaining stirring. When the temperature reached 60 °C, the hydrogel was poured into the mold and placed in the refrigerator at a temperature of 6 °C for 20 min to finalize the gelation process. Finally, the sample was removed from the mold and stored overnight (minimum 12 h) in distilled water in the refrigerator. Before each manipulation, the sample was returned to room temperature (around 19 °C) for at least one hour. To avoid dehydration and, hence, alteration of the phantom’s properties, the phantom was removed from the water just before the measurements and repositioned in the water no more than 10 min after its removal [32].

Depending on the experiment, the samples could be homogeneous or bilayer. For example, in the case of the preparation of a bilayer phantom with 2% and 5% agar mass concentration, a first 2% agar hydrogel was prepared according to the method described above and poured into the mold until half-full. During the gelation process in the refrigerator, the second 5% agar hydrogel was prepared with the same method and poured into the rest of the mold.

### 2.2. Impact-Based Analysis Method (IBAM)

**Figure 1:**
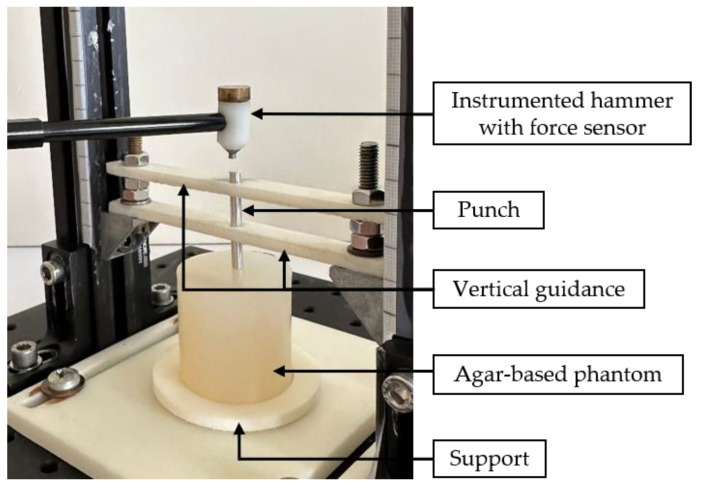
Experimental set-up of the impact-based analysis method (IBAM). During a measurement, the instrumented hammer impacts the punch, which is vertically guided. The lower part of the punch is in contact with the agar-based phantom held by the support.

The experimental set-up for the mechanical characterization method by impact analysis was described in Poudrel et al. [15] and is shown in Figure 1. Briefly, it consists of a 5-g hammer, equipped with a force transducer (type 8204, Brüel and Kjær (B&K), Naerum, Denmark) impacting a 35 mm long and 4 mm diameter cylindrical aluminum punch in contact with the upper surface of the phantom to be characterized. As shown in Figure 1, the system was integrated into a support that holds the agar sample and guides the punch vertically during the measurements. Note that this positioning system has been improved (i) in terms of phantom positioning through a standardized procedure, (ii) punch guidance with reduction in punch friction and (iii) impact properties thanks to rigidity of the set-up compared to Poudrel et al. [15].

**Figure 2:**
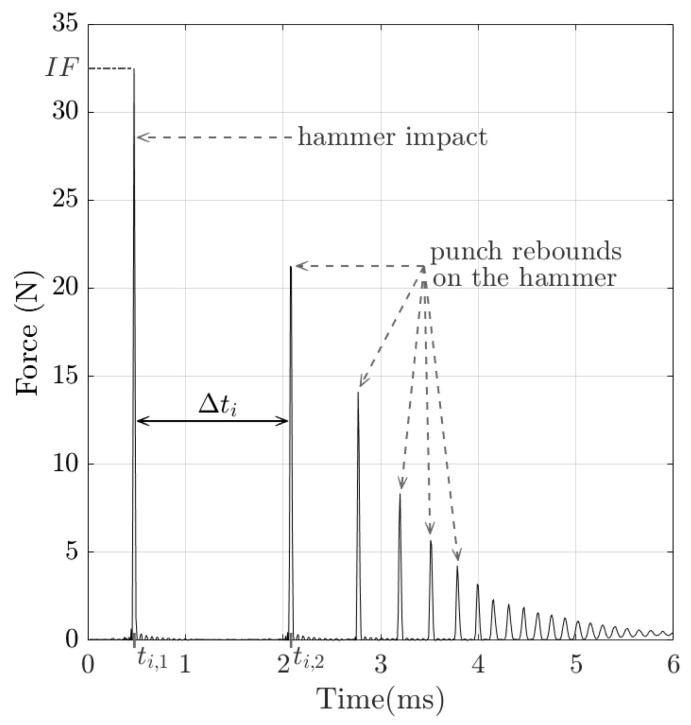
Example of a signal recorded by the force sensor impacting the hammer (impact *#i*) with a 3% agar-based phantom mimicking soft tissues. The temporal indicator $\Delta t_{i}$, where $t_{i,1}$ is the time of impact hammer and $t_{i,2}$ is the time of the first rebound of the punch on the hammer, and the impact force IF are indicated. Here, $\Delta t_{i}$ = 1.61 ms and IF = 32.5 N.

The variation in the force as a function of time obtained during the impact of the instrumented hammer on the punch was measured as the output data of the force transducer using LabVIEW software (version 15.0, National Instruments, Austin, TX, USA) and a data acquisition module with a sampling frequency of 102.4 kHz (NI 9234, National Instruments, Austin, TX, USA). A typical signal is shown in Figure 2. The first peak corresponds to the initial impact of the hammer on the punch, denoted by $t_{i,1}$, and the following peaks are related to the rebounds of the punch between the hammer and the phantom [15,22]. The maximum value of the first peak is referred to as impact force IF in what follows. Similarly, the time of the first rebound, corresponding to the second peak, is denoted by $t_{i,2}$.

A post-processing method adapted from Poudrel et al. [15,22] was applied to signals with an IF comprised between 30 and 35 N, which will be discussed in Section 4.2.1. The indicator $\Delta t_{i}=t_{i,2}-t_{i,1}$ was then determined for each impact *#i*.

Finally, the indicator Δt is defined by

(2) $$\Delta t=\frac{1}{5}\sum_{i=1}^{5}\Delta t_{i},$$

which corresponds to the average value of five consecutive impacts. Different numbers of impacts were tested to improve measurement repeatability, and the choice of 5 impacts was made as a compromise between the improvement of the results and measurement time. For each sample, five values of Δt were determined in order to assess the reproducibility of the measurements, which led to a total of 25 impacts having an IF comprised between 30 N and 35 N. Eventually, the average and standard deviation values of all five measurements of Δt were determined for each sample.

### 2.3. MyotonPro^®^

**Figure 3:**
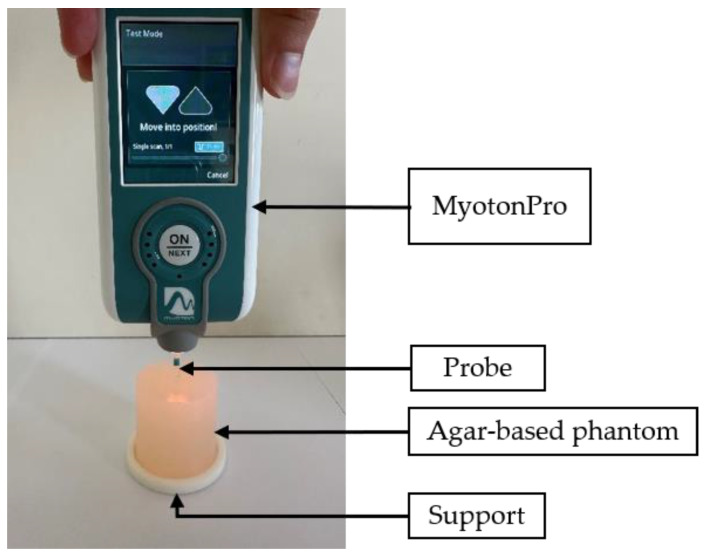
Experimental set-up using MyotonPro device to characterize a 3% agar-based phantom mimicking soft tissues.

As shown in Figure 3, MyotonPro (MyotonAS, Tallinn, Estonia) is a commercial handheld digital palpation device used for non-invasive characterization of soft tissues such as muscles, tendons, fascia or skin [33]. We followed the measurement process prescribed by the manufacturer, which consists of positioning the device with the standard probe perpendicular to the sample. Then, the tool applied a pre-compression force of 0.18 N followed by a mechanical pulse of 0.4 N for 0.15 ms. The tissue response was recorded using an accelerometer. After post-processing of the acceleration, velocity and displacement signals, five parameters (the oscillation frequency, the dynamic stiffness, the logarithmic decrement, the mechanical stress relaxation time and the ratio of relaxation and deformation time) were estimated to define the viscoelastic properties of the soft tissue [34,35]. In this study, only the dynamic stiffness S, defined by the manufacturer as the resistance of the tissues to a force of deformation produced by the mechanical pulse, was recorded. S is given by [33]

(3) $$S=\frac{a_{max}\times m_{probe}}{\Delta l},$$

where $a_{max}$ is the vertical acceleration at the instant of maximum contact compression force, Δl is the displacement at the same instant and $m_{probe}$ is the mass of the probe. For each sample, 15 measurements were realized with the test mode. The pulse time was equal to 0.07 ms [13] to enable measurements of all mass concentrations of agar-based phantoms. The average and standard deviation values of S were determined.

### 2.4. Dynamic Mechanical Analysis

**Figure 4:**
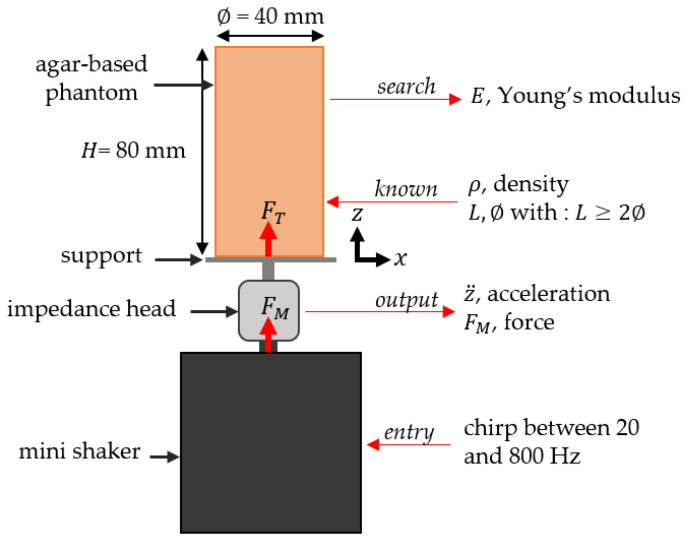
Schematic illustration of the custom vibration-based set-up performed on agar-based phantoms mimicking soft tissues to determine their Young’s moduli.

A custom vibration-based set-up [36] was applied to 5 cylindrical samples (length: 80 mm and diameter: 40 mm) with agar mass concentrations of 1%, 2%, 3%, 4% and 5% in order to assess their respective Young’s moduli. The system consists of a mini shaker (type 4810, B&K, Naerum, Denmark) connected to an impedance head (type 8001, B&K, Naerum, Denmark), onto which is screwed a support for the agar sample. The measurement protocol consists of imposing a displacement with the shaker under the form of a chirp function between 20 and 800 Hz with a peak-to-peak amplitude equal to 0.015 mm. The system’s responses in force $F_{M}\left(t\right)$ and acceleration $\ddot{z}\left(t\right)$ were recorded in the z direction using the impedance head, as shown in Figure 4.

As described in Ewins [37] and Silva et al. [38], the output of the impedance head had to be corrected because the mass of the support could not be neglected compared to the mass of the sample. The force sensor of the impedance head records a force $F_{M}\left(t\right)$, which writes [37]

(4) $$F_{M}\left(t\right)=F_{T}\left(t\right)+F_{app}\left(t\right),$$

where $F_{T}\left(t\right)$ is the force transmitted to the sample, and $\;F_{app}\left(t\right)$ is the force applied to the support of the sample. The transfer functions $Y_{M}\left(f\right)$ and $Y_{corr}\left(f\right)$ are defined as

(5) $$Y_{M}\left(f\right)=\frac{\hat{\ddot{z}}\left(f\right)}{{\hat{F}}_{M}\left(f\right)}\;\mathrm{and}\;Y_{corr}\left(f\right)=\frac{\hat{\ddot{z}}\left(f\right)}{{\hat{F}}_{T}\left(f\right)},$$

where ${\hat{F}}_{M}\left(f\right)$, ${\hat{F}}_{T}\left(f\right)$, ${\hat{F}}_{app}\left(f\right)$ and $\hat{\ddot{z}}\left(f\right)$ are the Fourier transforms of $F_{M}\left(t\right)$, $F_{T}\left(t\right)$, $F_{app}\left(t\right)$ and $\ddot{z}\left(t\right)$, respectively. The same custom vibration-based set-up is performed without any sample, which leads to the determination of $F_{M_{no-load}}\left(t\right)$ and ${\ddot{z}}_{no-load}\left(t\right)$ and to their respective Fourier transforms, ${\hat{F}}_{M_{no-load}}\left(f\right)$ and ${\hat{\ddot{z}}}_{no-load}\left(f\right)$. The spectral apparent mass $m_{app}\left(f\right)$ is then obtained following [39,40]

(6) $$m_{app}\left(f\right)=\frac{{\hat{F}}_{M_{no-load}}\left(f\right)}{{\hat{\ddot{z}}}_{no-load}\left(f\right)}.$$

${\hat{F}}_{app}\left(f\right)$ can then be expressed as

(7) $${\hat{F}}_{app}\left(f\right)=m_{app}\left(f\right)\times \hat{\ddot{z}}\left(f\right),$$

Following the correction method described in Cakar et al. [39] and Keswick et al. [40], it was possible to express the corrected transfer function $Y_{corr}\left(f\right)$ from the transfer function taken at the output of the impedance head, $Y_{M}\left(f\right)$ and the spectral apparent mass, $m_{app}\left(f\right)$ following

(8) $$Y_{corr}\left(f\right)=\frac{\hat{\ddot{z}}\left(f\right)}{{\hat{F}}_{T}\left(f\right)}=\frac{\hat{\ddot{z}}\left(f\right)}{{\hat{F}}_{M}\left(f\right)-m_{app}\left(f\right)\times \hat{\ddot{z}}\left(f\right)}\;\Rightarrow Y_{corr}\left(f\right)=\frac{Y_{M}\left(f\right)}{1-m_{app}\left(f\right)\times Y_{M}\left(f\right)}.$$

By modeling the agar-based phantom as a linear elastic rod beam of length L = 80 mm and diameter ∅ = 40 mm, which is excited by an imposed displacement at its lower end (at z = 0) and is free upper face (at z = L), the Young’s moduli of the phantoms E can be determined by [41]

(9) $$E=4\rho {\left(Lf_{1}\right)}^{2},$$

where $f_{1}$ is the fundamental resonance frequency of $Y_{corr}\left(f\right)$, and ρ is the density of the agar-based phantom.

### 2.5. Characterization Protocols

Various phantoms were manufactured following the protocol described in Section 2.1 in order to compare the performances of the IBAM and MyotonPro. To do so, three series of experiments described hereafter were performed.

#### 2.5.1. Estimation of the Measured Volume of Interest (VOI)

The aim of the first series of experiments was to determine the volume of interest (VOI) to which the method is sensitive for both measurement techniques. To do so, we considered cylindrical phantoms with 3% agar and varying diameters and lengths. The maximum phantom dimensions were defined so that both methods were not sensitive to boundary conditions. First, several phantoms with a constant length of 40 mm and with diameters ranging from 10 to 60 mm were tested in order to determine the sensitivity of both techniques to the sample diameter. Second, another cylindrical phantom, with a diameter of 40 mm and a length of 50 mm was made. Both methods were first applied on this sample and then for successive decreasing lengths of the sample. To do so, the sample length was progressively reduced by a few millimeters using a cutting guide, and the new length was re-evaluated using a digital caliper. This procedure was reproduced until the sample length was equal to 1.6 mm, so that the axial measuring range of both measurement methods could be assessed.

#### 2.5.2. Axial and Lateral Resolutions Using Bilayer Configurations

**Figure 5:**
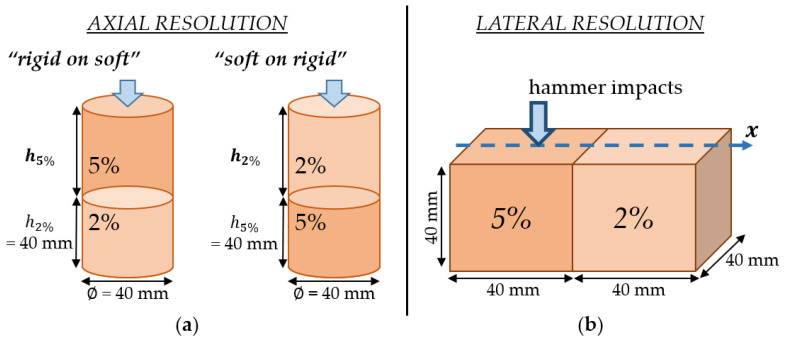
Illustration of the two experimental protocols for the estimation of the spatial resolution for the IBAM and MyotonPro. (**a**) Evaluation of the axial resolution for the two conditions “rigid on soft” and “soft on rigid” where the top layer thicknesses $h_{5\%}$ and $h_{2\%}$ (in bold on the figure) vary between 40 mm and 0 mm; (**b**) evaluation of the lateral resolution.

For the second series of experiments, the purpose was to evaluate the axial and lateral resolutions of the two methods following the protocol schematically described in Figure 5. To assess the axial resolution, two 80 mm long and 40 mm diameter cylindrical bilayer phantoms of 2% and 5% agar mass concentrations were considered. For the first configuration, the bottom layer of the phantom was 40 mm long and made of hydrogel with 2% agar, while the top layer thickness $h_{5\%}$ (made of 5% agar) was varied between 0 and 40 mm with a step comprised between 2 and 6 mm, as shown in Figure 5a. This configuration will be referred to as “rigid on soft” in what follows. For the second configuration, the bottom layer of the phantom was 40 mm thick and made of hydrogel with 5% agar, while the top layer thickness $h_{2\%}$ (made of 2% agar) was varied between 0 and 40 mm with a step comprised between 2 and 6 mm, as shown in Figure 5a. This configuration will be referred to as “soft on rigid” in what follows.

To assess the lateral resolution, bilayer phantoms of 2% and 5% agar, consisting of two 40 mm cubes placed side by side, were considered, one for each measurement technique. From these phantoms, several series of measurements were performed by translating the two devices along the x-axis passing through the upper surface of the phantom, defined by the blue line in Figure 5b. A measurement was taken every 1 mm using the IBAM and every 2 mm using MyotonPro to avoid being affected by the previous measurement.

#### 2.5.3. Stiffness Discrimination

The objective of the third series of experiments was to determine the effect of the agar mass concentrations, which is related to the stiffness, on the results obtained with both measurement techniques. To do so, five 40 mm long and 40 mm diameter cylindrical phantoms with agar mass concentrations of 1%, 2%, 3%, 4% and 5% were considered.

## 3. Results

### 3.1. Volume of Interest (VOI) of the Measurement

**Figure 6:**
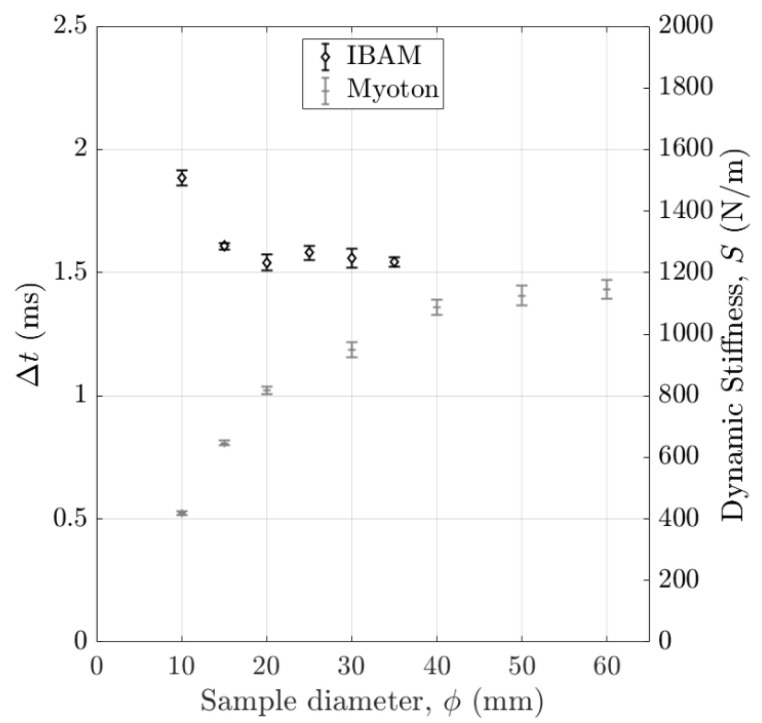
Variation in the indicators Δt and S obtained with the IBAM and MyotonPro as a function of the sample diameter with a 3% agar mass concentration. The error bars correspond to the reproducibility of the measurements.

Figure 6 shows the variations in the indicators Δt and S obtained with the IBAM and MyotonPro as a function of the sample diameter for a constant length of 40 mm. The error bars correspond to the reproducibility of the measurements. The indicator Δt stays approximately constant for diameters larger than 15 mm, while the indicator S still varies until around 40 mm.

**Figure 7:**
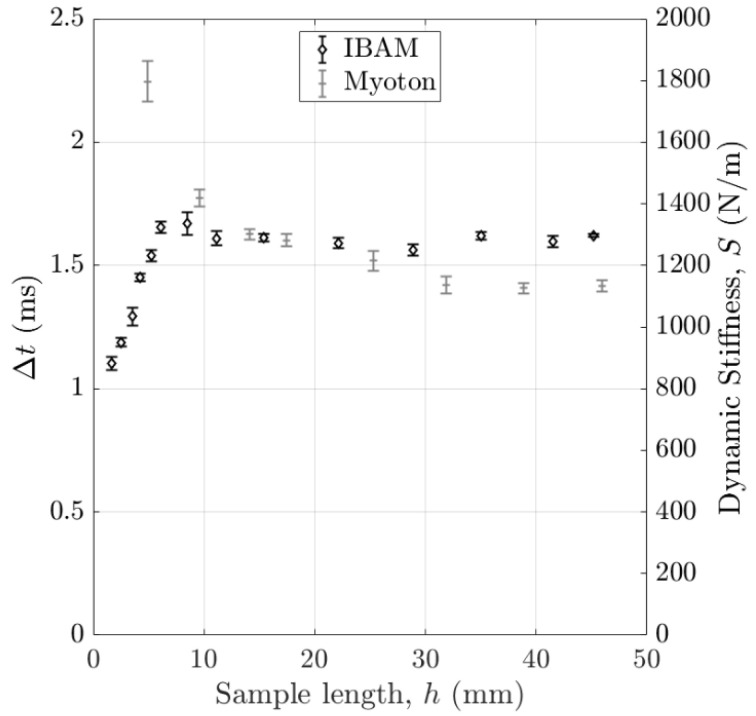
Variation in the indicators Δt and S obtained with the IBAM and MyotonPro as a function of the sample length with a 3% agar mass concentration. The error bars correspond to the reproducibility of the measurements.

Figure 7 shows the variations in the indicators Δt and S obtained with the IBAM and MyotonPro as a function of the sample length for a constant diameter of 40 mm. The error bars correspond to the reproducibility of the measurements. The indicator Δt stays approximately constant for lengths larger than 5 mm, while the indicator S still varies until around 30 mm.

These two results indicate that the VOI of the IBAM is smaller than that of MyotonPro, so that the IBAM is less sensitive to the sample size compared to MyotonPro.

### 3.2. Axial Resolution

**Figure 8:**
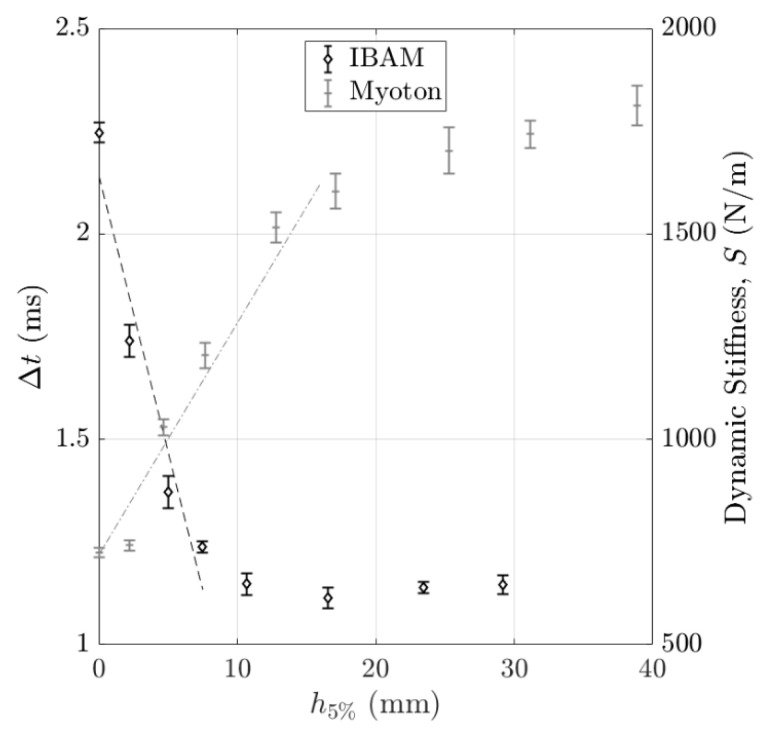
Variation in the indicators Δt and S obtained with the IBAM and MyotonPro as a function of the top layer thickness $h_{5\%}$ for the “rigid on soft” configuration. The error bars correspond to the reproducibility of the measurements.

Figure 8 shows the variations in the indicators Δt and S obtained with the IBAM and MyotonPro as a function of $h_{5\%}$ for the “rigid on soft” configuration. The error bars correspond to the reproducibility of the measurements. The indicator Δt stays constant for values of $h_{5\%}$ larger than 10 mm, while the indicator S stays approximately constant for $h_{5\%}$ larger than 25 mm. Note that for both indicators, the values obtained for $h_{5\%}$ = 0 and for $h_{5\%}$ = 40 mm are similar to the ones obtained with samples with mass concentrations of 2% and 5%, respectively. In Figure 8, the black dashed line (respectively, the gray dash-dotted line) is the linear regression of Δt (respectively, S) as a function of $h_{5\%}$, indicating the slope $a_{5}^{\Delta t}$ (respectively, $a_{5}^{S}$) between $h_{5\%}$ = 0 mm and $h_{5\%}$ = 8 mm (respectively, between $h_{5\%}$ = 0 mm and $h_{5\%}$ = 20 mm). For both methods, the values of slopes $a_{5}^{\Delta t}$ and $a_{5}^{S}$ will be given and exploited in Section 4.2.4 “Axial and lateral resolution”.

**Figure 9:**
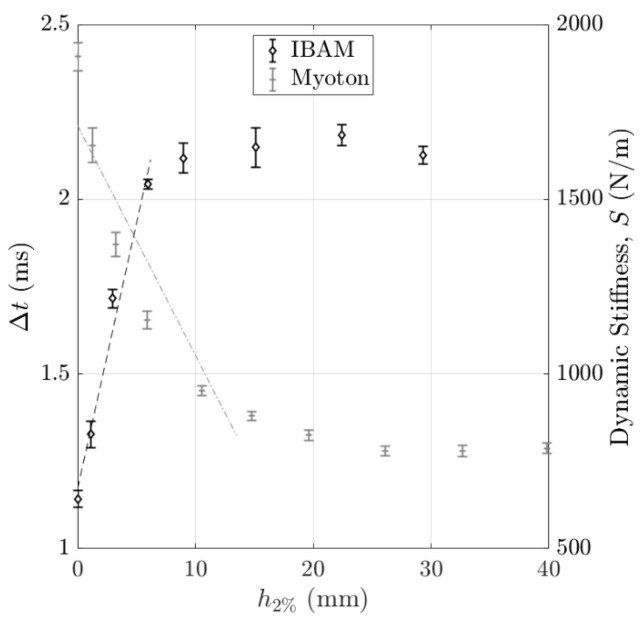
Variation in the indicators Δt and S obtained with the IBAM and MyotonPro as a function of the top layer thickness $h_{2\%}$ for the “soft on rigid” configuration. The error bars correspond to the reproducibility of the measurements.

Figure 9 shows the variations in the indicators Δt and S obtained with the IBAM and MyotonPro as a function of $h_{2\%}$ for the “soft on rigid” configuration. The error bars correspond to the reproducibility of the measurements. The indicator Δt remains constant for values of $h_{2\%}$ larger than 9 mm, while the indicator S remains constant for values of $h_{2\%}$ larger than 25 mm. Note that for both indicators, the values obtained for $h_{2\%}$ = 0 and for $h_{2\%}$ = 30 mm are similar to the ones obtained with samples with mass concentrations of 5% and 2%, respectively. In Figure 9, the black dashed line (respectively, the gray dash-dotted line) is the linear regression of Δt (respectively, S) as a function of $h_{2\%}$, indicating the slope $a_{2}^{\Delta t}$ (respectively, $a_{2}^{S}$) between $h_{2\%}$ = 0 mm and $h_{2\%}$ = 8 mm (respectively, between $h_{2\%}$ = 0 mm and $h_{2\%}$ = 15 mm). For both methods, the values of slopes $a_{2}^{\Delta t}$ and $a_{2}^{S}$ will be given and exploited in Section 4.2.4 “Axial and lateral resolution” of the Section 4.

### 3.3. Lateral Resolution

**Figure 10:**
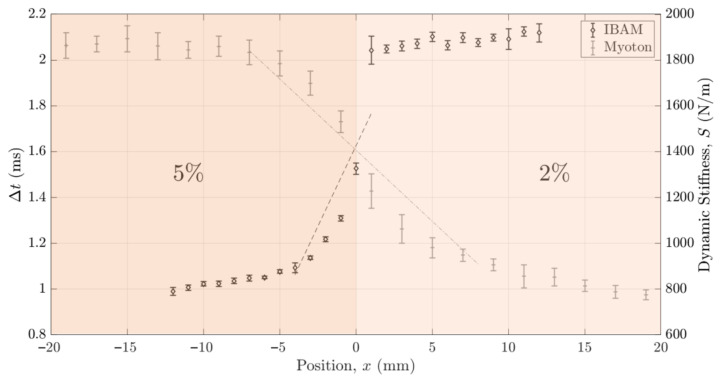
Variation in the indicators Δt and S obtained with the IBAM and MyotonPro as a function of the measurement position x on the upper surface of the bilayer samples. The error bars correspond to the reproducibility of the measurements. The color corresponds to the color indicated in Figure 5b.

Figure 10 shows the variations in the indicators Δt and S obtained with the IBAM and MyotonPro as a function of the position x of the device compared to the bilayer samples. The origin of the x-axis corresponds to the intersection between the two samples. The error bars correspond to the reproducibility of the measurements. The indicator Δt stays constant for values of x lower than −7 mm and larger than 2 mm, while the indicator S stays constant for values of x lower than −9 mm and larger than 15 mm. Note that the values obtained for both indicators for the minimal value of x and for the maximal value of x are similar to the ones obtained with samples with mass concentrations of 5% and 2%, respectively. In Figure 10, the black dashed line (respectively, the gray dash-dotted line) corresponds to the linear regression of Δt (respectively, S) as a function of x, leading to an estimation of the slope $a_{x}^{\Delta t}$ (respectively, $a_{x}^{S}$) between x = −5 mm and x = 1 mm (respectively, between x = −7 mm and x = 11 mm). For both methods, the values of slopes $a_{x}^{\Delta t}$ and $a_{x}^{S}$ will be given and exploited in Section 4.2.4 “Axial and lateral resolution” of the Section 4.

### 3.4. Stiffness Discrimination

**Figure 11:**
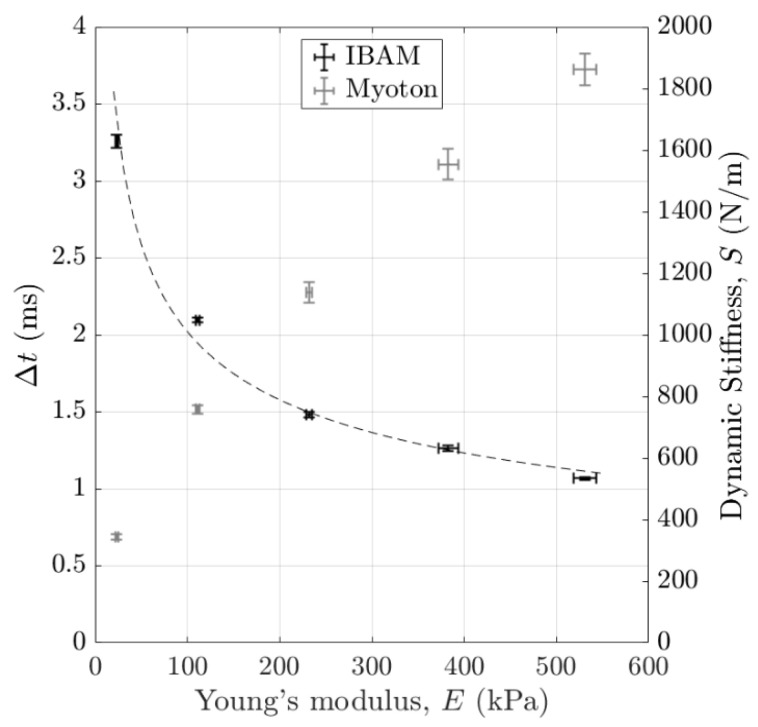
Variation in the indicators Δt and S obtained with the IBAM and MyotonPro as a function of the Young’s modulus E of soft tissue phantoms obtained for agar mass concentrations varying between 1 and 5% using custom vibration-based set-up. The error bars correspond to the reproducibility of the measurements. The dashed line is the curve fitting with a power function of Δt as a function of E.

Figure 11 shows the variations in the indicators Δt and S obtained with the IBAM and MyotonPro as a function of the sample Young’s modulus measured with the custom vibration-based set-up described in Section 2.4 for agar mass concentrations ranging from 1% to 5%. The error bars correspond to the reproducibility of the measurements. Both devices can discriminate between the five samples according to their stiffness. The Δt (respectively, S) is shown to decrease (respectively, increase) as a function of the sample Young’s modulus increases.

## 4. Discussion

The aim of this study was to demonstrate the performance of the impact-based analysis method (IBAM) to assess the stiffness of agar-based phantoms. Soft tissue phantoms were used to assess the performance of our method and compare it with those of an existing device, the MyotonPro [13]. The IBAM has the advantages of being non-invasive, real-time, quantitative, easy to use and inexpensive.

### 4.1. Young’s Modulus of Agar-Based Phantoms Mimicking Soft Tissues

**Figure 12:**
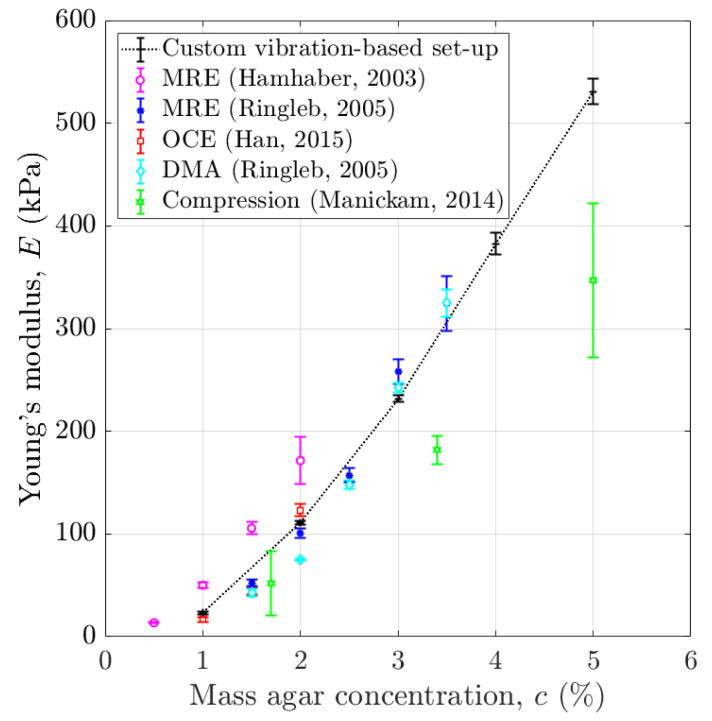
Comparison of the variation in the Young’s modulus of soft tissue phantoms E as a function of the agar mass concentrations using various measurement methods [26,42,43,44]. The error bars correspond to the reproducibility of the measurements.

The characteristics of agar hydrogels are very sensitive to preparation conditions and difficult to control [30,31]. For example, the elastic properties of various agar gels with the same mass concentration have been shown to vary by one or even two orders of magnitude as a function of agar mass concentration [30]. Figure 12 compares the variation in the sample Young’s modulus as a function of the agar mass concentration found herein with previous results obtained in the literature using elastography with magnetic resonance imaging (MRE) or with optical coherence tomography (OCE), quasi-static compression test or DMA [26,42,43,44]. Our results are shown to be consistent with other studies and to be acceptable to mimic healthy and abnormal soft tissue [1].

### 4.2. Physical Interpretation of Δt and S

#### 4.2.1. Effect of the Impact Force

As shown in Poudrel et al. [15], the average value of the indicator Δt as well as its reproducibility depend on the maximum value of the impact force IF. In order to optimize the reproducibility of the measurement, an impact was taken into account only when IF was comprised between 30 N and 35 N. As demonstrated in Poudrel et al. [15], the values of Δt decrease as a function of the impact force IF and tend to stabilize above 30 N, which explains the lower bound of the interval. Moreover, the value of IF had to be minimized to avoid damaging the samples, which explains the upper bound. The range of variation in IF (5 N) was chosen to be sufficiently large to be able to reproduce the impact manually but not too large to minimize the variability.

#### 4.2.2. Effect of the Sample Young’s Modulus

As shown in Figure 11, Δt decreases as a function of the Young’s modulus, which is consistent with the results found in Poudrel et al. [15]. This decreasing behavior has been explained qualitatively by the fact that Δt may be inversely related to the resonance frequency of the punch and phantom system, as shown in the context of other applications of the methods like osteotomies [22,25] or hip implant stability assessments [18,19,20,21]. Note that this behavior has been explained quantitatively using a 1D analytical model in Poudrel et al. [15]. Assuming that the hammer, the punch and agar-based phantom set-up can be modeled as a spring–mass system, the resonance frequency of the system can be simply modeled as being proportional to the square root of the Young’s modulus E of the agar-based phantom. Since Δt is inversely proportional to this resonance frequency, this assumption leads to

(10) $$\;\Delta t\propto E^{-0.5},$$

A logarithmic regression analysis of the data shown in Figure 11 and represented with the dashed line indicates that

(11) $$\;\Delta t\propto E^{-0.36},$$

Despite the simplicity of the model, which does not consider the geometry of the set-up nor the sample viscoelastic and non-linear behaviors, a good qualitative agreement is obtained with the experimental results.

The dynamic stiffness parameter S increases as a function of the sample Young’s modulus, which is also consistent with results realized with tissues mimicking phantoms [45] and biological soft tissues [46,47].

#### 4.2.3. Effect of the Volume of Interest (VOI)

The results shown in Figure 6 and Figure 7 indicate that the IBAM is not sensitive to changes in boundary conditions when located at a distance higher than 7.5 mm radially and 5 mm axially from the contact between the punch and the sample. These results indicate that for an agar mass concentration of 3%, the volume of interest (VOI) measured using the IBAM could be approximated by a hemi-sphere of radius approximately equal to 5 mm and centered on the center of the disc corresponding to the contact between the punch and the sample. However, these results may vary according to the agar mass concentration as well as on the diameter of the punch. Following a simple dimensional analysis, the hemi-sphere radius should be determined by the diameter of the punch since no other dimension intervenes in the system. The VOI measured using MyotonPro may be approximated by a hemi-sphere with a diameter around 30 mm, which indicates the lower VOI investigated by the IBAM (5 mm, see above). Note that another probe may be used with MyotonPro (arm L-shaped probe, MyotonAS, Tallinn, Estonia) to allow superficial assessment of soft tissue [48], but this probe leads to an estimation of shear tissue properties [49].

#### 4.2.4. Axial and Lateral Resolutions

In the case of bilayer phantoms, Δt and S are sensitive to both layers when the measurement is performed close to their interface. Determining the errors on the estimation of the distance between the position of the center of the punch and the interface may lead to a quantitative comparison of the axial and lateral resolutions of the IBAM and MyotonPro. To do so, for the “rigid on soft” configuration, a linear regression analysis, represented by the dashed line in Figure 8, was performed for Δt (respectively, S) by considering the interval where Δt (respectively, S) varies, so for $h_{5\%}$ lower than 10 mm (respectively, $h_{5\%}$ lower than 25 mm) (see Figure 8). The slope of the linear regression analysis obtained for Δt and S as a function of $h_{5\%}$ is noted as $a_{5}^{\Delta t}$ and $a_{5}^{S}$, respectively. Moreover, the average reproducibility of both measurement techniques was determined by averaging the standard deviation values obtained in the same intervals for Δt and S and was noted as $Err_{5}^{\Delta t}$ and $Err_{5}^{S}$. Following what has been performed in Vayron et al. [50,51], the error on the estimation of $h_{5\%}$ using both measurement methods reads

(12) $$\varepsilon_{5}^{\Delta t}=\left|\frac{Err_{5}^{\Delta t}}{a_{5}^{\Delta t}}\right|,\;\mathrm{for}\;\mathrm{the}\;\mathrm{IBAM}$$

(13) $$\varepsilon_{5}^{S}=\left|\frac{Err_{5}^{S}}{a_{5}^{S}}\right|,\;\mathrm{for}\;\mathrm{MyotonPro}$$

The same method was applied to the results about the “rigid on soft” configuration shown in Figure 9. We determined the slope of the linear regression analysis obtained for Δt and S as a function of $h_{2\%}$, noted as $a_{2}^{\Delta t}$ and $a_{2}^{S}$, by considering a linear regression analysis for both indicators Δt and S for $h_{2\%}$ lower than 9 mm and $h_{2\%}$ lower than 25 mm (see the dashed line in Figure 9), which led to the determination of the error on the estimation of $h_{2\%}$ noted as $\varepsilon_{2}^{\Delta t}$ and $\varepsilon_{2}^{S}$, respectively.

The same method was again applied to the results about the lateral resolution shown in Figure 10 concerning the lateral resolution. We determined the slope of the linear regression analysis obtained for Δt and S as a function of x, noted as $a_{x}^{\Delta t}$ and $a_{x}^{S}$ by considering a linear regression analysis for both indicators Δt and S for x between −7 mm and 2 mm and, respectively, for x between −9 mm and 15 mm, (see the dashed line in Figure 10), which led to the determination of the error on the estimation of x noted as $\varepsilon_{x}^{\Delta t}$ and $\varepsilon_{x}^{S}$, respectively.

Table 1 shows the results obtained for the values of the reproducibility, the slope and the estimation error for the three aforementioned configurations with the IBAM and MyotonPro. The values of the estimation error of $h_{5\%}$, $h_{2\%}$ and x obtained with the IBAM are shown to be significantly lower than the values obtained with MyotonPro. The results indicate the better sensitivity of the IBAM compared to MyotonPro. These results could be a step forward in the development of a future decision support system to delimit margins in the case of the resection of diseased tissue with different mechanical properties.

#### 4.2.5. Stiffness Sensitivity Comparison

Following the same approach for the estimation error as the one described above, we also compared the performances of the IBAM with those of (i) the custom vibration-based set-up and (ii) MyotonPro to assess the sample stiffness by analyzing the results shown in Figure 11, which is a simple way of determining the sensitivity of each method to changes in agar mass concentrations.

Because of the non-linear behavior of Δt and S as a function of the agar mass concentration, two ranges of variations were considered: (i) between 1% and 2% and (ii) between 1% and 5%. To do so, a linear regression analysis was performed with the data shown in Figure 11 for both indicators Δt and S and both ranges of variation, respectively.

The slopes of the linear regression analysis obtained for Δt and S are noted as $a_{E}^{\Delta t}$ and $a_{E}^{S}$, respectively. Moreover, the average reproducibilities of both measurement techniques were determined by averaging all standard deviation values obtained in the corresponding range of variation and noted as $Err_{E}^{\Delta t}$ and $Err_{E}^{S}$. Following what has been conducted in Vayron et al. [50,51], the error on the estimation of the E using both measurement methods reads

(14) $$\varepsilon_{E}^{\Delta t}=\left|\frac{Err_{E}^{\Delta t}}{a_{E}^{\Delta t}}\right|,\;\mathrm{for}\;\mathrm{the}\;\mathrm{IBAM}$$

(15) $$\varepsilon_{E}^{S}=\left|\frac{Err_{E}^{S}}{a_{E}^{S}}\right|,\;\mathrm{for}\;\mathrm{MyotonPro}$$

The same method was applied to the results obtained with the custom vibration-based set-up using our samples and with the literature data obtained with elastography and DMA for other agar-based phantoms [26,42,43,44] (see Figure 12). This analysis led to the determination of the error on the estimation of E using the aforementioned methods. Due to scarce data obtained in the literature [26,42,43,44], we could not consider both intervals for each dataset.

As shown in Table 2, the lowest error on the estimation of the sample stiffness in the range 1–2% is obtained using the custom vibration-based set-up, while this error (similar results are obtained herein and in the literature [44]) is similar to the error using the IBAM when considering the entire concentration range (1–5%). However, custom vibration-based set-up and DMA are difficult to be applied in vivo. Meanwhile, the IBAM has similar sensitivity to MyotonPro in the range 1–2% but has a significantly better sensitivity (i) compared to MyotonPro in the range 1–5% and (ii) compared to all other techniques. Note that the IBAM is much simpler and cheaper compared to elastography.

### 4.3. Limitations and Perspectives

First, we chose to use MyotonPro^®^ as the only device to establish a benchmark for comparison with the IBAM because it was shown to be the most effective for surface mechanical characterization of soft tissue [52]. However, in the future, the IBAM could also be compared with other surface stiffness measurement tools such as the Shore Durometer^®^ (Type 1600-OO, Rex Gauge, Brampton, ON, Canada) [13,52,53] or the IndentoPro^®^ (Fascia Research Group, Ulm University; Department of Human Movement Sciences, University of Chemnitz, Germany) [52,54]. Note that we did not use the Cutometer^®^ (Courage and Khazaka, Köln, Germany) [6,7,12] because after preliminary tests, no measurement could be performed using our agar-based phantoms.

Second, the reproducibility of hammer impacts on the punch could be improved, for example, in terms of punch guidance or hammer motion, thanks to a possible automation of the impact. Moreover, even if the duration of the measurement with agar-based phantoms lasted less than 10 min, new samples that are more stable over time, more durable and with standardized mechanical properties could be used to improve the methodology and reproducibility of the measurements. Nevertheless, only a quasi-static Young’s modulus without dissipative effects was taken into account in the mechanical characterization of the phantoms, and the viscoelastic effects of agar-based samples [26,31] was neglected.

Third, numerical modeling of the IBAM based on the model developed in Poudrel et al. [15] or using a finite element model could allow to study in more detail the determinant of Δt and to investigate the presence of other potential information, such as viscosity or non-linear geometrical/material effects.

## 5. Conclusions

This study investigates the performance of an impact-based analysis method (IBAM) to assess the biomechanical properties of phantoms mimicking soft tissues, which is compared with the results obtained with MyotonPro^®^. A temporal indicator Δt is derived from the force signal, resulting from the impact between the instrumented hammer and a punch in contact with the sample. Different homogeneous and bilayer agar-based samples were used to assess the spatial (axial and lateral) resolution together with the sensitivity to changes in Young’s moduli of the IBAM and of MyotonPro. The performances of the two aforementioned techniques were compared with those of DMA and elastography (MRE and OCE). The IBAM was shown to have a volume of interest (VOI) affecting the results equivalent to a hemi-sphere of diameter approximately twice the diameter of the punch, lower than that of MyotonPro. Moreover, the IBAM was shown to be more sensitive to stiffness variation than MyotonPro or elastography and to be equivalent to DMA. The IBAM has the advantage of being easy to use, cheap, real-time, objective and non-invasive. This study paves the way for the development of a decision support system for diagnosis, quantitative monitoring and evaluation of skin treatments for clinicians. A perspective could consist of a preclinical study in animals in order to demonstrate its applicability in vivo.

## Figures and Tables

**Table 1:** Results of the interface distance estimation error, for the axial and lateral resolutions, with the IBAM and MyotonPro. a denotes the slope of the linear fit of the result, Err denotes the average reproducibility and ε indicates the sensitivity of the measurements.

Interface Distance Estimation Error (mm)		Axial Resolution		Lateral Resolution
		“Rigid on Soft”	“Soft on Rigid”	
**Method**		$h_{5\%}$	$h_{2\%}$	x
Δt, impact-based analysis method (IBAM)	$a^{\Delta t}$ (ms/mm)	−0.13	0.15	0.14
	$Err^{\Delta t}$ (ms)	0.03	0.03	0.02
	$\varepsilon^{\Delta t}$ (mm)	0.22	0.17	0.15
S, MyotonPro	$a^{S}$ (N/m × mm)	56.51	−65.77	−62.17
	$Err^{S}$ (N/m)	25.33	29.39	49.21
	$\varepsilon^{S}$ (mm)	0.45	0.45	0.79

**Table 2:** Performances of different mechanical characterization methods (IBAM, MyotonPro, custom vibration-based set-up, DMA, elastography with magnetic resonance imaging (MRE) and with optical coherence tomography (OCE)) assessed with samples with different agar mass concentrations. a denotes the slope of the linear fit of the result as a function of Young’s modulus, Err denotes the average reproducibility and ε indicates the sensitivity of the measurements.

Experimental Data ^1^		Agar Mass Concentration	$a_{E}^{\Delta t}$	$Err_{E}^{\Delta t}$	$\varepsilon_{E}^{\Delta t}$
			ms/kPa	ms	kPa
Δt, impact-based analysis method (IBAM)		1% to 2%	−0.013	0.03	2.22
		1% to 5%	−0.003	0.02	5.42
			$a_{E}^{S}$	$Err_{E}^{S}$	$\varepsilon_{E}^{S}$
			N/m × kPa	N/m	kPa
S, MyotonPro		1% to 2%	4.73	10.86	2.29
		1% to 5%	2.94	31.28	10.65
			$a_{E}^{DMA}$	$Err_{E}^{DMA}$	$\varepsilon_{E}^{DMA}$
			-	kPa	kPa
Custom vibration-based set-up		1% to 2%	1	1.70	1.70
		1% to 5%	1	5.97	5.97
Literature data ^2^		agar mass concentration	$a_{E}^{Lit}$	$Err_{E}^{Lit}$	$\varepsilon_{E}^{Lit}$
			-	kPa	kPa
Elastography	DMA [44]	1% to 5%	1	5.30	5.30
	MRE [44]	1% to 5%	0.95	10.74	11.27
	MRE [42]	1% to 2%	0.92	7.98	8.71
	OCE [43]	1% to 2%	0.78	3.86	4.94

^1^ Results based on our experimental data presented in this article. ^2^ Results based on data from the literature.

## Data Availability

The data are stored in the MSME laboratory.

## Abbreviations

The following abbreviations are used in this manuscript:

IBAM	impact-based analysis method
DMA	dynamic mechanical analysis
MRE	magnetic resonance imaging
OCE	optical coherence tomography
VOI	volume of interest
